# MRI to delineate the gross tumor volume of nasopharyngeal cancers: which sequences and planes should be used?

**DOI:** 10.2478/raon-2014-0013

**Published:** 2014-07-10

**Authors:** Aron Popovtzer, Mohannad Ibrahim, Daniel Tatro, Felix Y. Feng, Randall K. Ten Haken, Avraham Eisbruch

**Affiliations:** 1 Department of Radiation Oncology, University of Michigan, Ann Arbor, USA; 2 Department of Radiology, University of Michigan, Ann Arbor, USA

**Keywords:** gross tumor volume, MRI, nasopharyngeal cancer, radiotherapy

## Abstract

**Background:**

Magnetic resonance imaging (MRI) has been found to be better than computed tomography for defining the extent of primary gross tumor volume (GTV) in advanced nasopharyngeal cancer. It is routinely applied for target delineation in planning radiotherapy. However, the specific MRI sequences/planes that should be used are unknown.

**Methods:**

Twelve patients with nasopharyngeal cancer underwent primary GTV evaluation with gadolinium-enhanced axial T1 weighted image (T1) and T2 weighted image (T2), coronal T1, and sagittal T1 sequences. Each sequence was registered with the planning computed tomography scans. Planning target volumes (PTVs) were derived by uniform expansions of the GTVs. The volumes encompassed by the various sequences/planes, and the volumes common to all sequences/planes, were compared quantitatively and anatomically to the volume delineated by the commonly used axial T1-based dataset.

**Results:**

Addition of the axial T2 sequence increased the axial T1-based GTV by 12% on average (p = 0.004), and composite evaluations that included the coronal T1 and sagittal T1 planes increased the axial T1-based GTVs by 30% on average (p = 0.003). The axial T1-based PTVs were increased by 20% by the additional sequences (p = 0.04). Each sequence/plane added unique volume extensions. The GTVs common to all the T1 planes accounted for 38% of the total volumes of all the T1 planes. Anatomically, addition of the coronal and sagittal-based GTVs extended the axial T1-based GTV caudally and cranially, notably to the base of the skull.

**Conclusions:**

Adding MRI planes and sequences to the traditional axial T1 sequence yields significant quantitative and anatomically important extensions of the GTVs and PTVs. For accurate target delineation in nasopharyngeal cancer, we recommend that GTVs be outlined in all MRI sequences/planes and registered with the planning computed tomography scans.

## Introduction

The introduction of intensity modulated radiation therapy (IMRT) has led to improvements in the treatment of nasopharyngeal cancer (NPC).^1^–^3^ Traditionally, the treatment of all gross tumor was secured through large lateral opposed radiation fields that included the base of the skull and adjacent tissues, with wide margins.^4^ By contrast, highly conformal techniques produce sharp dose fall-off gradients, sparing noninvolved tissues. Therefore, precise knowledge of the boundaries of the gross tumor volume (GTV) is crucial for defining the high-risk clinical target volume (CTV), and treatment must be carefully planned by imaging.

For evaluating tumor extent in advanced para-nasal sinus cancer and NPC, magnetic resonance imaging (MRI) has been found to be better than computed tomography (CT).^5^–^13^ It more accurately demonstrates base-of-skull involvement, intracranial extension, involvement of the prevertebral fascia, and subtle marrow space infiltration.^6^,^7^,^9^ Some studies have suggested that in patients with NPC and cranial nerve involvement, the more accurate contouring associated with MRI translates into a survival advantage.^8^ Accordingly, many radiologists rely on MRI scans for determination of target volumes for purposes of radiotherapy.^14^–^19^

However, the optimal MRI dataset (imaging sequences) that should be used for planning radiotherapy has not been established. For tumor staging, studies emphasize the importance of axial T1-weighted images acquired with fat suppression, both with and without gadolinium contrast, in addition to T2-weighted images and the use of coronal and sagittal acquisition planes.^8^ Although some authors found that the coronal imaging planes added important information regarding the cranial extent of tumors^7^,^19^, others reported that enhanced and nonenhanced T1 sequences were sufficient.^20^

This issue is particularly important in modern treatment planning which increasingly supports some form of interactive or automated image registration. Many studies describing techniques in clinical series of IMRT for NPC, as well as the recent NPC protocol of the Radiation Therapy Oncology Group^20^, recommended the registration and fusion of the MRI image with the planning CT scan whenever possible. However, they did not detail the MRI dataset used.^1^,^14^,^15^,^21^ Although Emami *et al*.^6^, in a comparative study of MRI and CT-based GTV delineation, used all MRI sequences, other studies that provided any specifics about the MRI methods applied for GTV delineation and fusion with the planning CT typically used axial T1-weighted images alone.^22^

The aim of the present study was to determine if radiotherapy planning may be based solely on MRI axial T1 imaging or if information from all available MRI datasets is required for accurate target delineation.

## Patients and methods

### Patients and setting

The study group consisted of 12 patients treated for NPC in 2003–2007. The study was approved by the institutional review board (IRB). The planning CT scans and pre-therapy MRI images were collected for analysis. The disease was categorized pathologically according to the classification of the World Health Organization (WHO) as type 3 in 9 patients, type 2 in 2 patients, and type 1 in 1 patient. Staging of the primary tumor was done according to published guidelines^23^: 9 patients had stage T4 tumor, 1 had T3, and 2 had T2.

### Procedure

The original CT treatment-planning scans were obtained at 3-mm slice thickness, and Intravenous contrast was injected in all cases. Patients were immobilized, scanned, and treated in the supine position, with the aid of a thermoplastic mask. CT-based sagittal, coronal, and oblique reconstruction images were made with the in-house radiation planning system. MRI was performed with a 1.5 Tesla GE Magnet scanner (Milwaukee, WI) using the regular head coil. The procedure was performed in the supine position, but neither a mask nor external markers were used. The imaging protocol was designed to obtain maximum information within a 1–1.5 h total scan time. T2-weighted sequences (TR/TE/excitations: 2000–2500 ms / 80–90 ms / 3) and Tl-weighted sequences (TR/TE/excitations: 500–600 ms / 10–30 ms /2–4) were acquired by standard spin-echo technique. Acquisition parameters of the axial and sagittal images were as follows: slice thickness 4 mm, slice gap 1 mm, field of view 20 cm with an image matrix of 256 x 256; parameters of the coronal images were slice thickness 3 mm, slice gap 0.5 mm, field of view 20 cm with an image matrix of 256 x 256. Four MRI datasets were studied: T2-weighted images in the axial plane without fat suppression (T2-AX, n = 12); gadolinium-enhanced Tl-weighted images in the axial plane with (n = 10) and without (n = 2) fat suppression (T1-AX); gadolinium-enhanced T1-weighted images in the coronal plane (COR, n = 12); and unenhanced T1-weighted images in the sagittal plane (SAG, n = 11).

In order to minimize inter-observer differences, the GTVs of the primary tumors were defined on each of the 4 MRI datasets by consensus of a neuroradiologist and two head and neck radiation oncologists. To assess the reproducibility of the consensus-based contour delineation, the GTVs on all 4 datasets of 3 patients were redrawn by the same team 3 months after the first contouring session, without review or presentation of the original contours. The magnitude of change in GTV size and the overlapping volumes between contouring sessions were noted and averaged. The percentage difference in GTV was defined according to the formula, (GTV1-GTV2)/(GTV1+GTV2)/2 x 100, where GTV1 is the GTV outlined in the first session and GTV2 is the volume outlined in the second session, using the same MRI dataset.

### Image registration

The CT data served as the basis for registration of the MRI data. Registration of each dataset was achieved in each case using a mutual information rigid translation algorithm.^24^ Final registration was accomplished by simultaneously superimposing the intersection of the brain surface with the axial CT slices, with the reconstructed sagittal and coronal CT images as contours. The MRI brain surface was interactively translated and rotated through a series of 3-D MRI dataset coordinate transformations until satisfactory visual agreement between the MRI surface and the CT images was obtained. Particular attention was addressed to the fixed bony landmarks in the region: vertebral bodies of C1 and C2, hard palate, lateral and medial pterygoid plates, and the clinoid processes. In this manner, the GTV outlined on each MRI study was fused to the CT dataset for comparison of extension and overlap, both qualitatively (in terms of anatomical extent) and quantitatively (in terms of physical volume, after computation of encompassing and overlapping volumes).

### Composite GTVs

Composite GTVs were formed by spatially adding the individual GTVs obtained by each MRI sequence and the physical volume (in cubic centimeters) of each composite structure. First, the COR and SAG datasets were assessed to determine if they added information to the traditional axial T1 image primarily, if they revealed potential GTV extensions attributable to differences in the imaging plane. The value of each composite GTV obtained from each MRI sequence was calculated. Second, two or more GTVs were combined, and the total volume (totV) and the common volume (comV), defined as volumes overlapping all sequences, were calculated. Three conditions were tested: T1-AX and T1-COR; T1-AX, T1-COR and T1-SAG; T1-AX and T2-AX). The T1-based volumes (standard) were compared to the additional volumes defined by the union of the COR and/or SAG datasets to the totV. Thereafter, the axial T2 datasets were compared to the axial T1 datasets to investigate differences obtained in GTVs due to the different pulse sequences. A uniform 5-mm expansion of the GTVs was made to yield the corresponding CTVs, and an additional uniform 4-mm expansion was made to yield the PTVs of the primary tumors.

Anatomical assessment was performed by visual inspection of the axial and the reconstructed sagittal and coronal CT planes in each case, together with the different fused MRI–based GTVs. Any extension in any direction of more than 1 cm beyond the axial T1-based GTV borders, in which the volume would not be covered by the PTV, or similar underestimation, was considered potentially clinically significant.

GTVs outlined using different MRI sequences in the same patients were quantitatively compared by two-tailed paired t-tests. Statistical significance was set at p < 0.05.

## Results

### Reproducibility

The reproducibility of the GTV delineation was examined in each MRI sequence/plane in 3 patients. There was an average change of 1.5% between the re-drawn GTVs (GTV2s) and the initial drawings (GTV1s) (Table 1). Examination of the anatomical overlap between the GTV1s and GTV2s showed that the envelope (union) of GTV1 and GTV2 averaged 10% larger than the GTV1 (Table 2), and the overlap (intersection) of GTV1 and GTV2 averaged 90% of the GTV1.These results were considered to indicate satisfactory reproducibility.^25^,^26^

### GTV and PTV statistics

**FIGURE 1.**
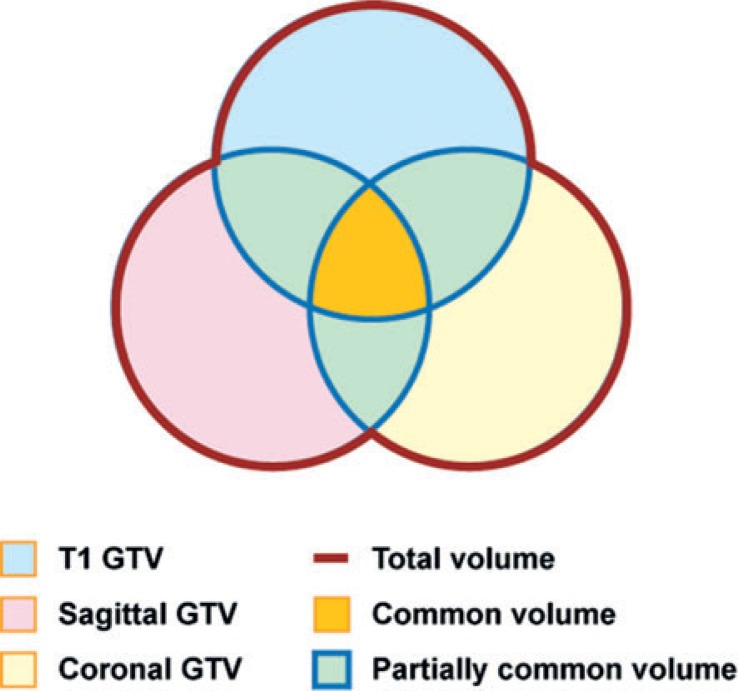
Differences among MRI sequences/planes. **(A)** Comparisons of gross tumor volume (GTVs) delineated by axial T1, axial T1+T2, and total axial T1+ sagittal + coronal T1 images for each patient. **(B)** Comparisons of total volume and axial T1-defined GTVs for each patient.

**FIGURE 2.**
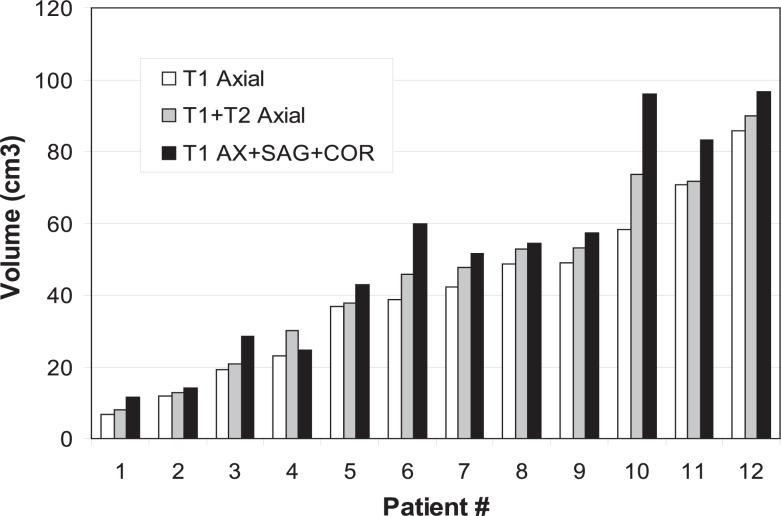
Comparisons of adding axial T2-defined GTVs to axial T1-defined gross tumor volume (GTVs) for each patient.

A summary of the GTV statistics for the T1 imaging planes is presented in Table 2 and Figures 1 and 2. In all cases, the T1-AX GTVs were larger than the GTVs of the other sequences/planes, and the addition of both the COR and SAG studies yielded an extension of the GTV beyond that defined by the T1-AX dataset alone (Figure 1). On average, addition of the COR GTVs increased the T1-AXl GTVs by 21% (SD = 18) (p = 0.005). Further addition of the SAG GTVs increased this combined volume by 10% (SD = 6) of the original T1-AX volume (p = 0.003), for an average combined increase of 30% over the original T1-AX volume. On average, 38% of the extended GTV volumes were common to all T1 sequences (Table 2).

A summary of the GTV statistics for the addition of T2-AX images to the T1-AX images is presented in Table 3 and Figure 2. On average, the GTVs derived from the T2-AX images increased the T1-AX GTVs by 12% (SD = 9) (p = 0.004).

A summary of the PTV statistics for all patients and all T1 imaging planes is presented in Table 4. As the PTVs were geometric expansions of the GTVs, the results were similar, but the percentage changes were smaller. On average, the addition of the COR PTVs increased the T1-AX PTVs by 14% (SD = 13) (p = 0.002), and the further addition of the SAG PTVs increased the combined volumes by 6% (SD = 4) (p = 0.04). On average, 50% (SD = 10) of the extended PTV volumes were common to both AX sequences and the COR and SAG sequences.

### Volume increase relative to T stage

There was no substantial difference between advanced and early local tumors in the increase in GTVs with the addition of various MRI sequences to the T1-AX sequence. In the 10 patients with T3-4 disease, the addition of SAG and COR-based volumes extended the T1-AX-based volumes by an average of 27% (SD = 20). The addition of T2-Ax-based GTVs extended the T1-based GTVs by 12% (SD = 9.8). In the 2 patients with T2a disease, the addition of the Cor- and SAG-based GTVs increased the T1-AX-based GTVs by 20% and 48%, and the addition of the T2-AX-based GTVs increased the T1-AX-based GTVs, by 18% and 8%.

### Anatomical assessments

**FIGURE 3.**
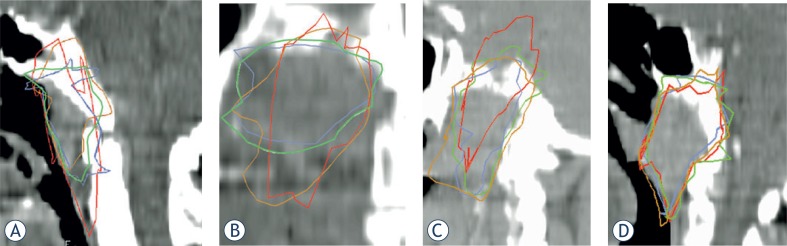
Gross tumor volume (GTVs) based on different MRI sequences fused into sagittal **(A, C, D)** or coronal **(B)** CT images. Brown = sagittal-based GTV; blue = axial T2-based GTV; red = coronal -based GTV; and green = axial T1-based GTV.

Table 5 shows the findings of the anatomical assessments of the extensions/ underestimations of the GTVs delineated by various MRI sequences or by T1-AX sequences alone. Representative cases are illustrated in Figures 3A-D. Addition of the COR-based images to the T1-AX sequences led to an extension of the caudal border of the GTV in 3 cases (Figure 3B) and of the cranial border in 2 cases; however, the COR images underestimated the posterior or anterior extent in 4 cases and the medial extent in 1 case (Table 5). Addition of the SAG images led to an extension of the superior and inferior borders of the GTVs in 2 cases (Figures 3A, 3C). Addition of the T2-AX images generally led to nonspecific extensions (in terms of direction) into soft tissue regions which were more subtly visualized than on the T1-AX images.

There were 3 cases in which volumes based on different sequences and planes were almost identical qualitatively (Table 5, Figure 3D). In these cases, the comV:totV ratio was between 42% and 48%, and the addition of the COR and SAG images extended the T1-AX-based volume by 12–16%.

Anatomically, the superior extensions of the COR- and SAG-based GTVs led to the identification of cavernous sinus, brain, and clivus involvements in which subtle involvement was not appreciated on axial MRI (Figures 3A, 3C). Drawing the GTVs in the COR and SAG planes also aided in extending the targets caudally into muscles that were not included in the T1-AX-based GTVs (Figures 3B, 3C).

## Discussion

In the radiology literature, there is a consensus regarding the necessity of performing MRI for staging nasopharyngeal tumors, and several authors have examined the benefits of different MRI sequences in this setting.^6^,^7^,^27^ However, unlike the diagnostic radiologist who uses MRI to assess the extent of the gross tumor for staging purposes, the radiation oncologist requires an accurate definition of the edges of the radiological abnormalities in order to define the GTV in 3 dimensions as reliably as possible. To the best of our knowledge, the present study is the first to assess in detail the utility of various MRI sequences/planes in defining the GTVs of NPC. The results show that when the GTV data from the axial T2 sequence and coronal and sagittal planes were added to the gadolinium-enhanced axial T1 sequence, the size and extent of the GTVs increased significantly. It has been proven that there is a correlation between volume of tumor and outcome^28^, and therefore the changes might have been were large enough to have clinical importance as well. Notably, the main changes were in the caudal and cranial direction, including cavernous sinus involvement which was occasionally missed when the axial dataset was used alone. We suggest that as we do not know which dataset represents the “true” extent of the GTVs, defining a composite GTV that encompasses the targets outlined using all datasets is the safest approach to defining the target for highly conformal radiotherapy of NPC. Our study suggests that if only axial images are available, the GTVs may be underestimated. Adding coronal and sagittal MRI images is expected to substantially improve target delineation. It is reasonable to continue the current study by analyzing the impact of MRI on radiation volume assessment when tumor delineation is performed in all three planes with the use of T1 sequence and fat suppression after administration of contrast. This will be the subject of future study.

These findings support previous diagnostic radiology studies, such as those of Chung *et al*.^7^ and Sakata *et al*.^19^, which suggested that coronal imaging plays a unique role in detecting cranial, base of skull, and clivus tumor extension. However, they disagree with the study of Lau *et al*.^20^ which suggested that coronal, sagittal and axial T2-weighted images do not have an impact on tumor staging and are therefore redundant. We found that sagittal plane-based GTVs significantly increased the volume obtained by the coronal and axial-plane MRI, whereas Vogl *et al*.^27^ stated that for staging purposes, the sagittal views had little impact and could be omitted. Furthermore, although Ng *et al*.^9^ suggested that for smaller tumors, relying only on axial T1 images is sufficient for staging purposes, our findings suggest that the addition of the coronal and sagittal views has a similarly important impact on GTV estimation in both early-stage and locally advanced tumors.

Previous studies of GTV measurements in head and neck cancers, including NPC, suggested prominent interobserver differences and smaller though still considerable intraobserver differences.^25^,^26^ To limit this problem in our study, the GTV was derived by consensus among several observers. The reproducibility of GTV delineation by the consensus members, as tested in our study, was reasonable, and the differences noted in the reproducibility test were substantially smaller than the differences in the GTVs that were related to the different MRI datasets.

This study was limited by the need to register MRI and CT scans made at different head and neck positions. The tight head and neck coil used for the clinical MRI scans did not accommodate the immobilization system used for the planning CT. The registration of MRI-based GTVs with the planning CTs using landmarks in the skull and base of the skull was previously found to be highly reliable for brain tumors and for tumors residing near the base of the skull, and this technique has been used clinically in our department for many years. However, the caudal-most extension of the tumors, beyond the base of the skull, may have not been well registered, potentially leading to an error in our volumetric calculations. Nevertheless, the most important message of our study is that different MRI sequences may produce different extensions of the GTV cranially, toward the cavernous sinus and related anatomical structures, which may not be appreciated using the axial T1 sequence alone.

The ability to register non-axial MRI images into the axial planning CT dataset, as performed in this study, is available in the in-house treatment planning system used at our institution. Some commercial planning systems do not have this capability, although in many cases, manufacturers have come out with new versions that do (Bruce Curran, PhD, personal communication). Our results suggest that this capability should be used for multi-sequence delineation of GTVs of tumors near the base of the skull.

PTVs were obtained in this study by uniform expansions of the GTVs. As expected, there was a lesser difference between the axial T1 and multiple MRI sequences for PTVs than GTVs. We speculate that as PTV margins are tightened by daily localization and correction of the set-up uncertainties^29^,^30^, the benefits of multi-sequence MRI for PTVs will be close to the ones found for GTVs.

In conclusion, using MRI for defining the primary NPC GTV, fusion of GTVs delineated on axial T1 sequences with coronal and sagittal images as well as with T2 sequences significantly changes the sizes and extents of the GTVs calculated on the basis of gadolinium-enhanced T1 axial images alone. These differences may have a major impact on GTV definition in some cases. Our results suggest that incorporating all MRI datasets in GTV delineation should be routine clinical practice in highly conformal radiation therapy.

## Figures and Tables

**TABLE 1.** Volume Percentage differences between the initial GTVs and the re-drawn GTVs for 3 cases for all sequences (average difference across all planes/sequences = 1.5%)

**% Diff .GTV SIZES**				

	**Sag (%)**	**T1 (%)**	**Cor (%)**	**T2 (%)**
**PATIENT 1**	4.1	1.9	−0.5	−3.6
**PATIENT 2**	−0.4	9.5	−4.3	−4.3
**PATIENT 3**	−8.2	15.4	0.1	8.3
**Sequence Avg.**	−1.5	8.9	−1.6	0.1

**TABLE 2.** GTVs derived from different T1 imaging planes

	**Axial T1 (cm3)**	**COR T1 (cm3)**	**SAG T1 (cm3)**	**Total T1 (A+C+S) (cm3)**	**Total T1 (% of AX)**	**Common T1 (A&C&S) (cm3)**	**Common T1 (% of Total)**
**Mean**	40.9	34.7	30.4	51.7	130.1%	21.2	38.3%
**Range**	7–86	6–76	6–60	12–97	107–170	3–47	18–55
**STD**	23.6	21.6	19.7	29.3	22.5%	14.0	10.9%

**TABLE 3.** GTVs derived from different T1 and T2 Axial imaging planes

	**Axial T1 (cm3)**	**Axial T2 (cm3)**	**Total (T1+T2) (cm3)**	**Total (T1+T2) (% of T1 AX)**	**Common (T1&T2) (cm3)**	**Common (T1&T2) (% of Total)**
**Mean**	40.9	32.7	45.3	112.3%	28.3	62.6%
**Range**	7–86	7–63	8–90	101–130	6–57	51–71
**STD**	23.6	21.6	25.1	9.1%	15.8	6.7%

**TABLE 4.** PTVs derived from different T1 imaging planes

	**Axial T1 (cm3)**	**COR T1 (cm3)**	**SAG T1 (cm3)**	**Total T1 (A+C+S) (cm3)**	**Total T1 (% of AX)**	**Common T1 (A&C&S) (cm3)**	**Common T1 (% of Total)**
**Mean**	136.0	117.5	103.6	160.9	120.1%	81.4	50.0%
**Range**	43–234	44–221	38–191	71–280	106–149	28–139	37–68
**STD**	57.7	52.6	49.4	68.9	16.7%	38.1	10.0%

**TABLE 5.** Anatomical GTV extents based on various MRI sequences compared with the axial T1-based GTVs

**Direction of Extension**	**Coronal MRI**	**Sagittal MRI**
Cranial	2+	2+
Caudal	3+	2+ & 2−
Medial	1−	2−
Lateral		3−
Posterior	3−	
Anterior	1−	

+ = No. of patients where the GTV border extended beyond the T1 axial GTV;

− = No. of patients where the GTV border underestimated compared to the T1 axial GTV (By at least 1 cm in each direction)
